# Appendiceal Mucocele Presenting as a Right Adnexal Mass: A Case Report

**DOI:** 10.1155/2010/281053

**Published:** 2010-09-13

**Authors:** Grigor Gortchev, Slavcho Tomov, Dobromir Dimitrov, Vasil Nanev, Tatyana Betova

**Affiliations:** ^1^Gynecologic Oncology Clinic, Medical University, Pleven, Bulgaria; ^2^Surgical Oncology Clinic, Medical University, Kliment Ohridski Street, 5800 Pleven, Bulgaria; ^3^Department of General and Clinical Pathology, Medical University, Pleven, Bulgaria

## Abstract

A 68-year-old female presented to the Gynecologic Oncology Clinic with a right-lower quadrant abdominal mass 3 × 4 cm in diameter palpable on pelvic examination. Her routine laboratory tests were normal. Transvaginal ultrasonography revealed a cystic mass in the right adnexa 3.9 cm in diameter, which was thought to arise in the ovary. At the time of laparoscopy, a 3 × 4 cm tumor arising from the distal end of the appendix was noted. A laparoscopic appendectomy with tumor removal was performed. Histologic examination of the surgical specimen revealed a mucocele of the appendix (AM). Although rare, this tumor should be considered in the differential diagnosis of a right adnexal mass. These tumors can be identified laparoscopically and removed by minimally invasive surgery.

## 1. Introduction

 Appendiceal mucocele (AM) is a rare entity that can present in a variety of ways. The incidence of AM is estimated to be 0.2-0.3% of appendectomy specimens.

Appendiceal mucocele was first described by Rokitanski in 1842, and the term refers to dilatation of the appendiceal lumen by an abnormal accumulation of mucus [1].

Mucoceles are histologically subdivided into four types based on the following classification introduced by the World Health Organization [2]:

1. retention mucocele or obstructive form of mucocele defined as cystic dilatation of the distal appendix with accumulation of abnormal mucoid material in the appendiceal lumen secondary to appendiceal outflow obstruction,
2. mucinous cystadenoma defined as a dilated mucus/mucin-filled appendix containing adenomatous mucosa lined by atypical mucinous epithelium containing basal nuclei and showing only minimal dysplastic features,
3. mucinous cystadenocarcinoma defined as adenocarcinoma associated with mucus-filled cystic dilatation of the appendix presenting as a mucocele,
4. myxoglobulosis defined as cystic dilatation of the appendix associated with mucinous globoid bodies.

## 2. Case Report

**Figure 1:**
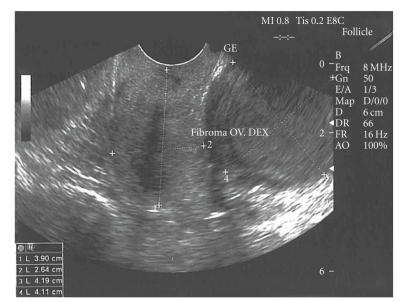
Intravaginal ultrasonography shows a 3.9 mm cyst mass in the right lower abdominal quadrant.

**Figure 2:**
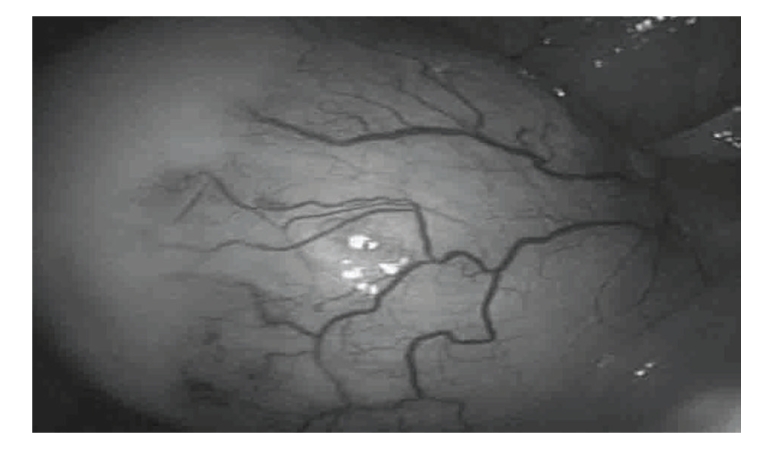
Laparoscopic view of appendiceal mucocele.

**Figure 3:**
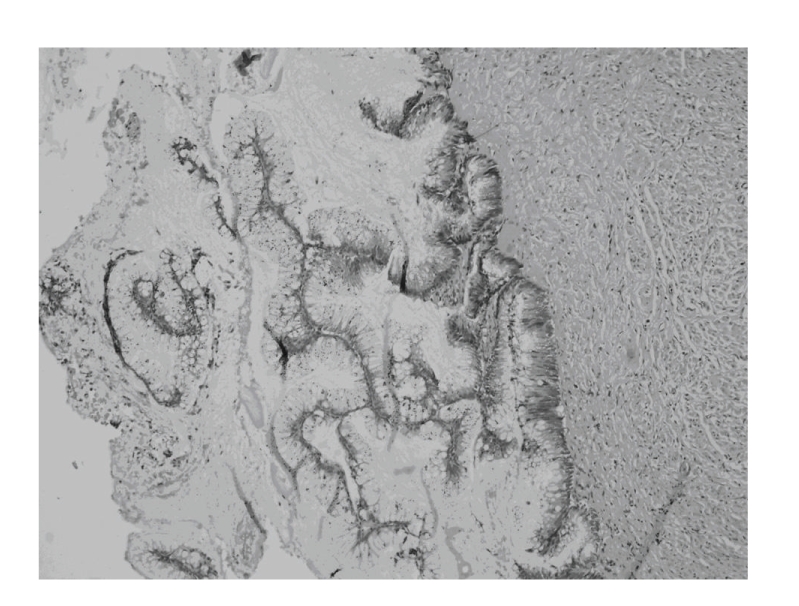
Histopathological microphotograph of the accumulated mucin in the appendix lumen.

 A 68-year-old postmenopausal female presented to the Gynecologic Oncology Clinic with a two-day history of spotting. She did not report any abdominal pain. Her medical history was significant in that she was hypertensive and had adult-onset diabetes mellitus. Her abdomen was soft and nondistended, with normoactive bowel sounds. Bimanual palpation in the lower-right abdominal quadrant revealed a tumor mass 4 cm in diameter. It was slightly mobile and had smooth walls. This mass was confirmed on pelvic and rectal examinations. The patient's laboratory tests were as follows: hemoglobin 10.9 g/l, white blood cells 5.2 × 10.3, blood glucose 6.2 mmol/l, blood urea 8.3 mmol/l, and serum creatinine 98 cmol/l. Her urinalysis was normal. Transvaginal ultrasonography revealed a cystic mass in the right lower abdominal quadrant 3.9 cm in diameter (Figure 1). This mass was interpreted sonographically as originating from the right ovary. There were no inflammatory changes or free fluid in the abdominal cavity. Endometrial biopsy revealed an atrophic endometrium. Diagnostic laparoscopy revealed bilateral atrophic ovaries, a normal-size uterus, and normal-appearing small bowel and liver. There was a 3 cm × 4 cm mass originating from the apex of the vermiform appendix, located adjacent to the right ovary (Figure 2). A laparoscopic appendectomy was performed, and the specimen was placed in an endocatch bag and removed. There was no evidence of tumor rupture at the time of surgery. Histologic evaluation of the surgical specimen revealed a mucocele of the appendix (Figure 3). Following surgery, the patient had a normal clinical course and was discharged from the hospital on the third postoperative day in satisfactory condition.

## 3. Discussion

 Mucocele of the vermiform appendix can be confused radiologically with an ovarian tumor, which may prove to be a diagnostic challenge. In a woman presenting with a right adnexal mass, appendiceal tumors should be considered in the differential diagnosis [3].

Mucocele of the appendix is most likely to occur in the sixth or seventh decade of life in women. The most common presenting signs and symptoms of patients with this entity are right lower quadrant abdominal pain (27%), abdominal mass (16%), weight loss (10%), and change in bowel habits (5%) [1]. Complications of mucocele include intussusception, bleeding, perforation, peritonitis, rupture, and finally, pseudomyxoma peritonei [4].

Surgery is the treatment of choice and should be performed immediately so that appendiceal carcinoma can be ruled out as the causative factor for the mucocele. Early diagnosis and prompt treatment is important to avoid unintended rupture and the development of pseudomyxoma peritonei. Extensive laparoscopic dissection, traumatic grasping of the appendix, or transport of the specimen through the abdominal wall may contribute to peritoneal dissemination of mucin containing cells. These complications can be avoided by using atraumatic graspers to handle the mucocele and using a nonpermeable bag to deliver the specimen through the port [5]. Conversion to laparotomy should be considered if the tumor is ruptured, if the tumor clearly extends beyond the appendix, or if there is evidence of metastases [6].

## 4. Conclusion

 The diagnosis of mucocele of the appendix as well as other appendiceal neoplasms should be considered by the gynecologist in the differential diagnosis of a right-sided adnexa tumor. Laparoscopy can be used to diagnose this tumor, and to facilitate its surgical removal.
